# Treatment of Diphtheria

**Published:** 1878-01

**Authors:** 


Treatment of Diphtheria.—Our village contains less than six hundred inhabitants. Has long been noted for its healthfulness. About the middle of last September it was visited by an epidemic of diphtheria, since which time there have been sixteen deaths from the disease. All the deaths were between the ages of two and fifteen years. I have treated thirty cases in all, some of marked severity. I had one death only; it in a family of fourteen members, five of whom were subjects of the disease, crowded into a room twelve by eighteen feet, in squalor, filth and ignorance. This case had epistaxis till he nearly bled to death, then laryngitis, with exudation, till his life was despaired of; yet he lived two weeks after all traces of the diphtheretic membrane had disappeared. I feel satisfied that, had he had proper hygiene and suitable nursing and food, he would have recovered. I used in all but my first case the sulpho-carbolate of sodium, as recommended by Dr. Anthony, of Providence, R. I. I use it in doses from five to thirty grains, in an aromatic syrup. I also use freely a saturated solution of chlor. pot., beef tea and milk. No swabbing or gargle; tonics when indicated. I have noticed an entire absence of fetŏr in my cases, which is in marked contrast with cases treated by others, who have used quinine, tr. ferri. chlor., pot. chlor, etc. I have not seen the least bad effect from its use in large doses. I am beginning to have faith in it as a prophylactic, and think it is worthy of further trial.—*D. A. Philips, M.D., in Philadelphia Medical and Surgical Reporter.*

Another evidence of the carbolic acid treatment of diphtheria being useful.—[Ed.]

